# Efectividad y Riesgo de Recurrencia de la Radiofrecuencia Pulsada en Neuralgia del Trigémino y Cefalea en Racimos Crónica

**DOI:** 10.31083/RN51129

**Published:** 2026-05-28

**Authors:** Marta Domínguez, Alicia Gonzalez-Martinez, Sonia Quintas, Carmen Montero, Iris Fernández-Lázaro, Elena Rojo, Manuel Muñoz, Dolores Ochoa, Concepción Pérez, José Vivancos, Ana Beatriz Gago-Veiga

**Affiliations:** ^1^Unidad de Cefaleas, Servicio de Neurología, Hospital Universitario de la Princesa Instituto de Investigación Sanitaria, Princesa (IIS-Princesa), 28006 Madrid, España; ^2^Departamento de Medicina, Universidad Autónoma de Madrid, 28029 Madrid, España; ^3^Unidad del Dolor, Hospital Universitario de la Princesa Instituto de Investigación Sanitaria, Princesa (IIS-Princesa), 28006 Madrid, España

**Keywords:** radiofrecuencia pulsada percutánea, neuralgia del trigémino, cefalea en racimos, refractario, recurrencia, percutaneous pulsed radiofrequency, trigeminal neuralgia, cluster headache, refractory, recurrence

## Abstract

**Introducción:**

La radiofrecuencia pulsada percutánea (RPP) es una técnica de neuromodulación mínimamente invasiva empleada en dolor crónico. Nuestro objetivo fue evaluar su efectividad y seguridad en casos refractarios de neuralgia del trigémino (NT) y cefalea en racimos (CR) crónica.

**Métodos:**

Estudio retrospectivo de pacientes con NT y CR crónica según criterios tercera edición clasificación internacional cefaleas (ICHD-3), tratados con RPP (ganglio de Gasser en NT y ganglio esfenopalatino en CR) en un periodo de 8 años. Para evaluar efectividad se analizaron a los 3 meses: reducción del número de días con dolor en NT y número de ataques en CR, tasa de respuesta del 50%, escala visual analógica (EVA), impresión de mejoría global (PGI-I, *Patient Global Impression of Improvement*) y recurrencia del dolor.

**Resultados:**

Veinticinco pacientes cumplieron criterios de inclusión, 19 NT y 6 CR, con una edad de media de 76 y 52 años respectivamente. Se observó una mejoría en 14/19 (73,6%) pacientes con NT y en 5/6 (83,3%) con CR. Hubo una tasa de respuesta del 50% en 11/14 (78,6%) con NT y 4/6 (66,7%) con CR. Además, encontramos una reducción del EVA a los 3 meses en ambos grupos (*p* < 0,0001 en NT; *p *= 0,06 en CR). PGI-I fue mejor o mucho mejor en 10/19 (52,6%) NT y 5/6 (83,3%) CR. El dolor recurrió en 13/14 (92,8%) en NT y 5/5 (100%) en CR, fundamentalmente tras 1 año desde el primer procedimiento (7/13 NT y 2/5 CR). No se registraron eventos adversos importantes.

**Conclusiones:**

En nuestra serie, la RPP se ha mostrado como un tratamiento efectivo y seguro en NT y CR refractarias. Aunque el estudio es retrospectivo y la muestra limitada, especialmente en CR, estos hallazgos son preliminares pero clínicamente relevantes. A pesar de la recurrencia del dolor, dada su baja tasa de complicaciones, podría ser de elección en pacientes no candidatos a procedimientos quirúrgicos.

## Abstract (en)

**Introduction:**

Percutaneous pulsed radiofrequency (PPRF) is a minimally invasive neuromodulation technique used in chronic pain management. Our aim was to evaluate its effectiveness and safety in refractory cases of trigeminal neuralgia (TN) and chronic cluster headache (CH).

**Methods:**

We conducted a retrospective study of patients with TN and CH diagnosed according to the International Classification of Headache Disorders, 3rd edition (ICHD-3) criteria and treated with PPRF (Gasserian ganglion for TN and sphenopalatine ganglion for CH) over an 8-year period. Effectiveness at 3 months was assessed by evaluating reduction in the number of pain days in TN and number of attacks in CH, ≥50% response rate, visual analogue scale (VAS), Patient Global Impression of Improvement (PGI-I), and pain recurrence.

**Results:**

Twenty-five patients met the inclusion criteria, including 19 with TN and 6 with CH, with a mean age of 76 and 52 years, respectively. Clinical improvement was observed in 14/19 (73.6%) patients with TN and 5/6 (83.3%) patients with CH. A ≥50% response rate was achieved in 11/14 (78.6%) patients with TN and 4/6 (66.7%) patients with CH. VAS scores decreased at 3 months in both groups (*p* < 0.0001 in TN; *p* = 0.06 in CH). PGI-I was rated as “better” or “much better” in 10/19 (52.6%) patients with TN and 5/6 (83.3%) patients with CH. Pain recurrence occurred in 13/14 (92.8%) patients with TN and 5/5 (100%) patients with CH, mainly after 1 year following the first procedure (7/13 TN and 2/5 CH). No major adverse events were reported.

**Conclusions:**

In our series, PPRF appears to be an effective and safe treatment for refractory TN and CH. Although the study is retrospective and the sample size is limited, particularly in CH, these findings should be considered preliminary but clinically relevant. Despite the high recurrence rate, given its low complication profile, it may represent a treatment option for patients who are not candidates for surgical procedures.

## 1. Introducción

La radiofrecuencia es una técnica empleada como tratamiento en distintas patologías que cursan con dolor crónico, como puede ser el caso de algunas cefaleas refractarias. Consiste en aplicar una corriente eléctrica en el territorio nervioso donde se origina el dolor, y crear así un campo eléctrico [1] que produce el movimiento de iones en los tejidos, con un consecutivo bloqueo de las señales dolorosas [2]. La sensibilidad a la corriente aplicada es diferente en función del tipo de fibra (sensitivas-nociceptivas o no mielinizadas, o sensitivas- no nociceptivas y mielinizadas), con una mayor tolerancia a la temperatura en las fibras sensitivas mielinizadas, lo que permite la afectación exclusiva de las fibras nociceptivas [3].

Existen dos tipos principales de radiofrecuencia: convencional continua y pulsada. La radiofrecuencia convencional (RFC), en la que se aplica una estimulación eléctrica convencional, tiene como objetivo aumentar la temperatura hasta producir una lesión térmica irreversible en las fibras nerviosas nociceptivas y de esta manera interrumpir permanentemente las señales de dolor [1, 4]. Esta técnica presenta altas tasas de efectividad, aunque sus potenciales efectos adversos pueden ser mantenidos como por ejemplo la anestesia dolorosa o la xeroftalmia [5].

Por su parte, la radiofrecuencia pulsada percutánea (RPP), de desarrollo más reciente, se considera una técnica mínimamente invasiva, simple y rápida, con una selectividad muy alta para lograr el bloqueo de las estructuras nerviosas [6, 7]. Utiliza pulsos de 20 milisegundos de lesión y una pausa de 480 milisegundos, lo que evita un excesivo aumento de temperatura que pudiera ocasionar daño tisular permanente [4]. De hecho, y a diferencia de la RFC, la RPP no causa daños estructurales en las fibras nerviosas y presenta menores efectos secundarios [7]. Recientemente, se habría confirmado la ausencia de diferencias estadísticamente significativas en términos de efectividad entre ambas técnicas en diferentes patologías, por lo que actualmente algunos autores recomiendan la RPP por ser un procedimiento más seguro y con un menor riesgo de complicaciones [8].

La RPP se ha utilizado con éxito para el tratamiento de innumerables enfermedades que cursan con dolor, incluido el dolor radicular, dolor en articulaciones sacroilíacas, artropatía facetaria, dolor postquirúrgico o dolor craneofacial [9]. Entre las distintas topografías para el uso potencial de la RPP en el tratamiento del dolor craneofacial se encuentran el ganglio de Gasser en el tratamiento de la neuralgia del trigémino (NT) y el ganglio esfenopalatino (GSP) en la cefalea en racimos (CR) [3, 10].

Ambas patologías presentan limitaciones en su manejo terapéutico, especialmente en el caso de la CR crónica. Aunque en la NT existen múltiples opciones farmacológicas, un porcentaje relevante de pacientes en ambas entidades acaba siendo refractario a los tratamientos habituales, mostrando una necesidad terapéutica no cubierta [2, 11]. En este sentido, la RPP se postula como un tratamiento a considerar en estas entidades [12, 13]. Sin embargo, y debido a la baja prevalencia de la NT clásica y de la CR, existe escasa evidencia sobre la efectividad y seguridad de la RPP en la práctica clínica, sin que se hayan descrito posibles factores clínicos y/o sociodemográficos predictores de una respuesta positiva o negativa. En este estudio se incluyen ambas condiciones para describir la experiencia clínica en la práctica real; dado que la NT y la CR son entidades clínicas distintas, los resultados deben interpretarse de manera individual dentro de cada entidad.

El objetivo de nuestro estudio fue analizar la efectividad de la RPP en pacientes con diagnósticos de NT y CR atendidos en la Unidad del Dolor de un hospital terciario, así como el porcentaje de recurrencia y la seguridad de la técnica. Por último, quisimos evaluar la asociación entre factores clínicos y/o sociodemográficos y la respuesta al tratamiento.

## 2. Material y Métodos 

### 2.1 Población de Estudio

Estudio observacional retrospectivo, de pacientes con diagnóstico de NT clásica y refractaria, o cefalea en racimos crónica y refractaria (CRCR), según los criterios de la tercera edición clasificación internacional cefaleas (ICHD-3) [14], procedentes de la Unidad del Dolor de un hospital terciario a los que se había realizado RPP, aplicada sobre el ganglio de Gasser en los casos de NT y sobre el GSP en los casos de CRCR. Se realizó un muestreo no probabilístico consecutivo.

### 2.2 Criterios de Inclusión y Exclusión

Se incluyeron pacientes con diagnóstico de NT o CRCR según los criterios de la ICHD-3, tratados mediante RPP durante un periodo de recogida de datos de 8 años. En los pacientes con NT, la RPP se realizó sobre el ganglio de Gasser, mientras que en los pacientes con CRCR se realizó sobre el GSP.

Se excluyeron aquellos pacientes en los que existían dudas sobre el diagnóstico, con coexistencia de otras causas de dolor craneofacial, aquellos tratados con procedimientos intervencionistas distintos a la RPP realizados por la Unidad del Dolor, como ablación percutánea del ganglio de Gasser o RFC como primer procedimiento, los pacientes con un periodo de seguimiento inferior a un año, y aquellos en los que no se disponían datos suficientes para considerarlos elegibles para el estudio.

Los datos basales de los pacientes, incluyendo número de días con dolor en NT o frecuencia de ataques en CRCR, así como la información de seguimiento, se obtuvieron mediante revisión detallada y sistemática de las historias clínicas de los pacientes. Las historias fueron revisadas por un neurólogo especialista en cefaleas.

Pacientes que posteriormente recibieron cirugía de Janetta (en NT) o neuroestimulación (en algunos casos de CR) no fueron excluidos, dado que estas intervenciones se realizaron tras la RPP y forman parte del seguimiento de los pacientes incluidos.

### 2.3 Técnica de RPP 

La técnica de RPP empleada fue la siguiente:

Ganglio de Gasser: se realiza bajo sedación, con agujas 22 G de 10 centímetros y punta activa de 5 milímetros (cat. no. DHC-022/100/5, OWL RF Insulated Hybrid Cannula, Diros Technology Inc., Markham, Ontario, Canada). El paciente se coloca en decúbito supino y se dirige el haz de rayos de TC en dirección subcigomática y con 10–15º oblicuo ipsilateral hasta visualizar el foramen oval (vista submentoniana oblicua). La punta de la aguja se sitúa a unos 2 cm lateral a la comisura de la boca del lado ipsilateral. A continuación, se introduce la aguja hacia el agujero oval bajo fluoroscopia en tiempo real, primero en la vista submentoniana AP y luego en la vista lateral. Se inicia una estimulación motora a 0,5 V hasta que se observa contractura del masetero. Con contracción y a 0,2–0,3 V corresponde con V3, si se progresa más, el momento en que se pierde la contracción corresponde con V2. Para encontrar V1 se progresa en proyección lateral hasta llegar a la unión del petroso con el clivus. Se da una lesión de RPP por rama a 45 V durante 3 minutos.

Ganglio esfenopalatino: se realiza bajo anestesia local y sedación consciente, empleando agujas 22 G, de 10 centímetros y 5 milímetros de punta activa. El paciente se coloca en decúbito supino con la cabeza fijada a la mesa y se dirige el haz de rayos de TC en proyección lateral para alinear los bordes inferiores de los ángulos de la mandíbula y superponer las fosas esfenopalatinas. La inserción suele hacerse infracigomática, siendo nuestro objetivo el tercio superior de la fosa esfenopalatina. La aguja se introduce primero en la vista lateral y se avanza medial y superiormente hacia la fosa pterigopalatina mediante fluoroscopia intermitente. Una vez en la dirección adecuada, se obtiene una vista anteroposterior y se introduce la punta de la aguja en la fosa pterigopalatina anteroposterior, y se avanza la punta de la aguja hasta la pared nasal. La aguja debe estar situada por dentro de la línea nasal externa sobre el cornete medio. Se produce la estimulación sensorial para que el paciente perciba a <0,5 V parestesias en la región nasal. Se realizan también 3 minutos de RPP a 45 V.

### 2.4 Variables Incluidas en el Estudio

Para evaluar la efectividad, se estimó como variable de respuesta terapéutica la reducción del dolor tras la RPP. En pacientes con NT, se consideró la reducción en días con al menos un episodio lancinante típico, y no el número de descargas por día. En pacientes con CR, se consideró la reducción del número de ataques, siguiendo los criterios de la ICHD-3. La variable de respuesta se evaluó a los 3 meses tras el procedimiento.

Como variables secundarias se incluyeron la tasa de respuesta de al menos 50% a los 3 meses, el porcentaje de reducción en el consumo de analgésicos, el tiempo hasta la mejoría, el cambio en la puntuación en la escala visual analógica del dolor (EVA) (escala visual analógica del dolor) a los 3 y la puntuación en la escala PGI-I (*Patient Global Impression of Improvement*) a los 3 meses del procedimiento. La escala EVA es una escala que otorga al dolor una puntuación entre 0 y 10, siendo 10 el máximo dolor que se puede sentir y 0 la ausencia de dolor [15]. La escala PGI-I evalúa la respuesta al tratamiento en base a una escala de Likert con una puntuación del 1 al 7, correspondiendo el 7 a “ha empeorado muchiśimo” y el 1 a “ha mejorado muchísimo” [16].

Respecto a la recurrencia, se recogieron las variables recurrencia del dolor y tiempo hasta recurrencia. La recurrencia se definió como un aumento ≥50% respecto al valor alcanzado tras la RPP en la evaluación a los 3 meses, mantenido ≥1 mes y/o la necesidad de intensificar tratamiento o repetir RPP. Asimismo, en relación con la seguridad, se recogió la variable complicaciones intraoperatorias y/o post-operatorias.

### 2.5 Análisis Estadístico

Se realiza una estadística descriptiva de los datos de la muestra. Las variables cuantitativas que siguen una distribución normal según la prueba de Kolmogorov-Smirnov se presentan como medias y desviación típica, mientras que las variables con distribución asimétrica se presentarán mediante medianas y rangos intercuartílicos (RIQ), diferencia entre el tercer y el primer cuartil de una distribución. En el caso de las variables cualitativas se usarán frecuencias relativas y absolutas.

En el análisis exploratorio de asociación de variables clínicas y respuesta al 50% se han utilizado métodos estadísticos paramétricos o no paramétricos según la distribución de las variables. Se utilizaron las pruebas de chi-cuadrado de Pearson o el test exacto de Fisher para comparar variables cualitativas. Las variables cuantitativas se analizaron mediante la prueba *t* de Student o la U de Mann–Whitney según la distribución de los datos. Para la comparación de variables antes–después del tratamiento se emplearon pruebas pareadas (*t* de Student pareada o test de Wilcoxon signed-rank) en función de la distribución de las variables. Las pruebas estadísticas se realizarán con un nivel de significación del 5% (*p* < 0,05) y serán bilaterales. Se utilizará el paquete estadístico SPSS versión 21.0 (IBM Corporation, Armonk, NY, USA), para realizar el análisis.

## 3. Resultados

**Fig. 1.**
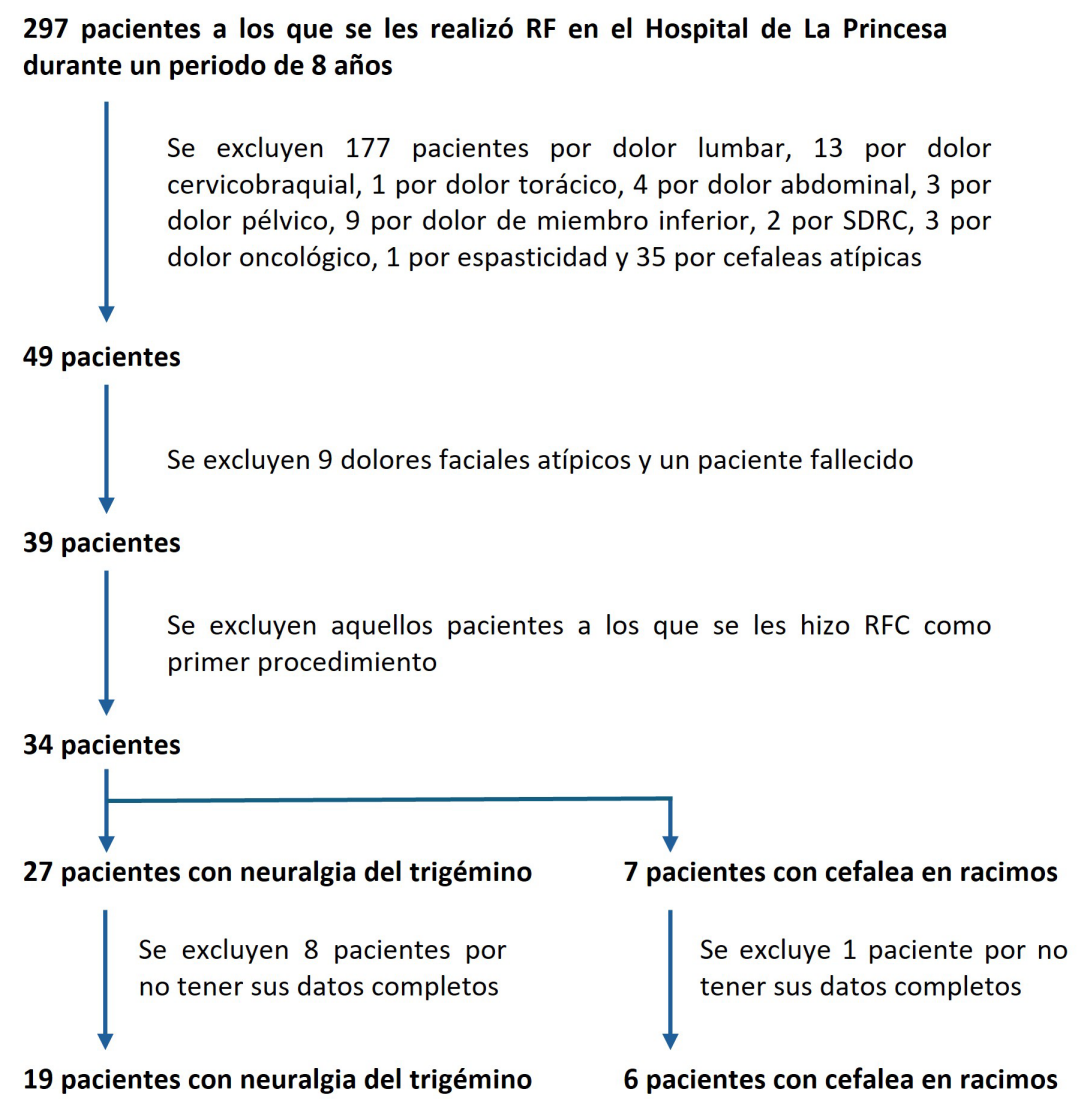
**Diagrama de flujo de los pacientes incluidos en el estudio**. RFC, radiofrecuencia convencional; SDRC, Síndrome de Dolor Regional Complejo.

De un total de 297 pacientes tratados mediante radiofrecuencia en nuestro centro durante el periodo de estudio, 25 pacientes cumplían los criterios de inclusión y ningún criterio de exclusión. El diagrama de flujo con la selección de los pacientes y los motivos de exclusión se incluye en la Fig. 1.

### 3.1 Descripción de la Muestra

Las características clínico-demográficas de los pacientes se encuentran resumidas en la Tabla 1. Los pacientes con NT en su mayoría fueron mujeres (68,4%) con una edad media en torno a 76,6 años y con una media de evolución de la neuralgia de 7 años. En el caso de CR, la mayoría fueron hombres (16,7%), con una edad media en torno a 52,2 años y un tiempo de evolución medio de 14 años. En ambos casos se habían empleado más de 4 fármacos preventivos previo al procedimiento.

**Tabla 1.** **Variables clínicas y demográficas de los pacientes con neuralgia del trigémino y cefalea en racimos tratados mediante radiofrecuencia pulsada percutánea (RPP)**.

Variables		Neuralgia del trigémino (N = 19)	Cefalea en racimos (N = 6)
Mujeres, n (%)		13 (68,4)	1 (16,7)
Edad en años, media (DE)		76,6 (11,6)	52,2 (18,6)
HTA, n (%)		12 (63,1)	1/6 (16,7)
DL, n (%)		9 (47,4)	1/6 (16,7)
DM, n (%)		1 (5,3)	0
Hábito tabáquico, n (%)		2 (10,5)	4/6 (66,7)
Trastorno del ánimo, n (%)		6 (31,6)	0
AP de otra patología que curse con dolor crónico, n (%)		1 (5,3)	0
Edad de inicio de la enfermedad (años), media (DE)		65,1 (12,8)	33,3 (15,5)
Edad de diagnóstico de la enfermedad (años), media (DE)		66,11 (11,7)	37,7 (15,8)
Tiempo de evolución de la enfermedad en 1^a^ RF (años), media (DE)		7,4 (5,2)	13,7 (13,0)
Nº fármacos preventivos usados, media (DE)		4,5 (1,4)	5,5 (2,2)
Estudio de imagen realizado			
	TC craneal, n (%)	2 (10,5)	2 (33,3)
	RM craneal, n (%)	17 (89,5)	4 (66,7)
Presencia de contacto vascular, n (%)		7 (36,8)	-

DE, desviación estándar; HTA, hipertensión arterial; DL, dislipemia; DM, diabetes mellitus; AP, antecedentes personales; RF, radiofrecuencia; TC, tomografía computarizada; RM, resonancia magnética.

### 3.2 Efectividad de la RPP

#### 3.2.1 Neuralgia del Trigémino

**Fig. 2.**
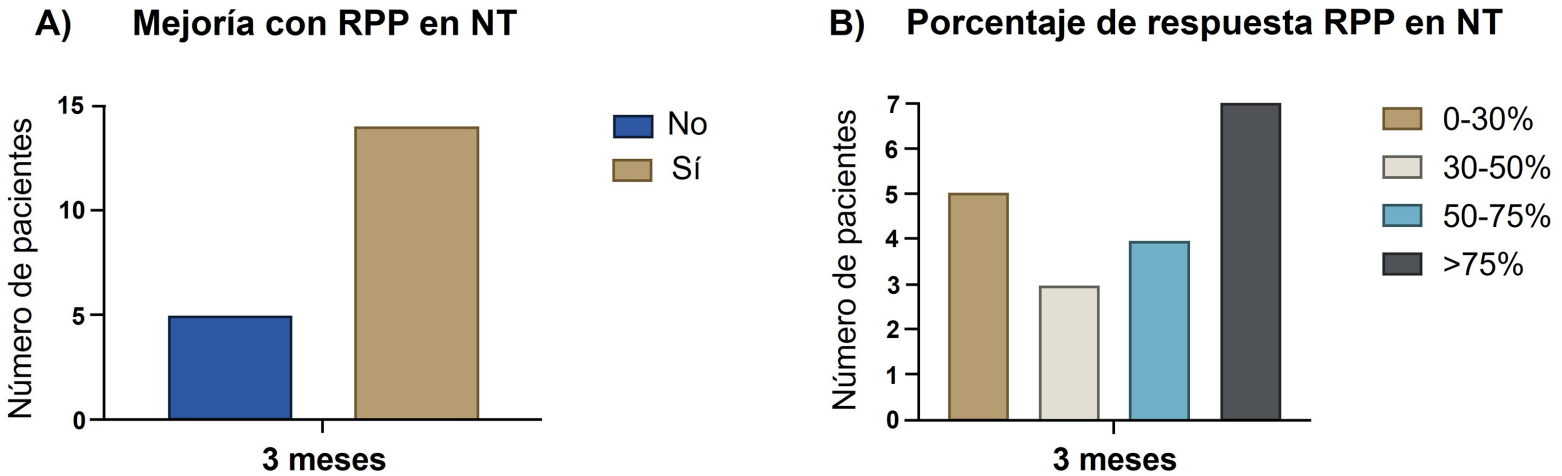
**Efectividad de la RPP en pacientes con neuralgia del trigémino**. (A) Pacientes que presentaron algún tipo de mejoría en el número de crisis. (B) Porcentaje de mejoría en el número de crisis mensuales. NT, neuralgia del trigémino.

Catorce de los 19 pacientes (73,6%) presentaron algún grado de mejoría en el número de días con dolor tras la radiofrecuencia. Los porcentajes de mejoría están representados en la Fig. 2. Se alcanzó una tasa de respuesta de al menos 50% en 11/14 (78,6%) de los pacientes que mejoraron.

Además, se observó una reducción en el consumo de analgésicos en 12/19 pacientes (63,1%). El tiempo hasta la mejoría en los pacientes que mejoraron fue de menos de 72 horas tras el procedimiento en 2/19 (10,5%), en 3/19 (15,8%) se evidenció entre 3 y 7 días después y en 4/19 (21,1%) entre una semana y un mes después.

**Fig. 3.**
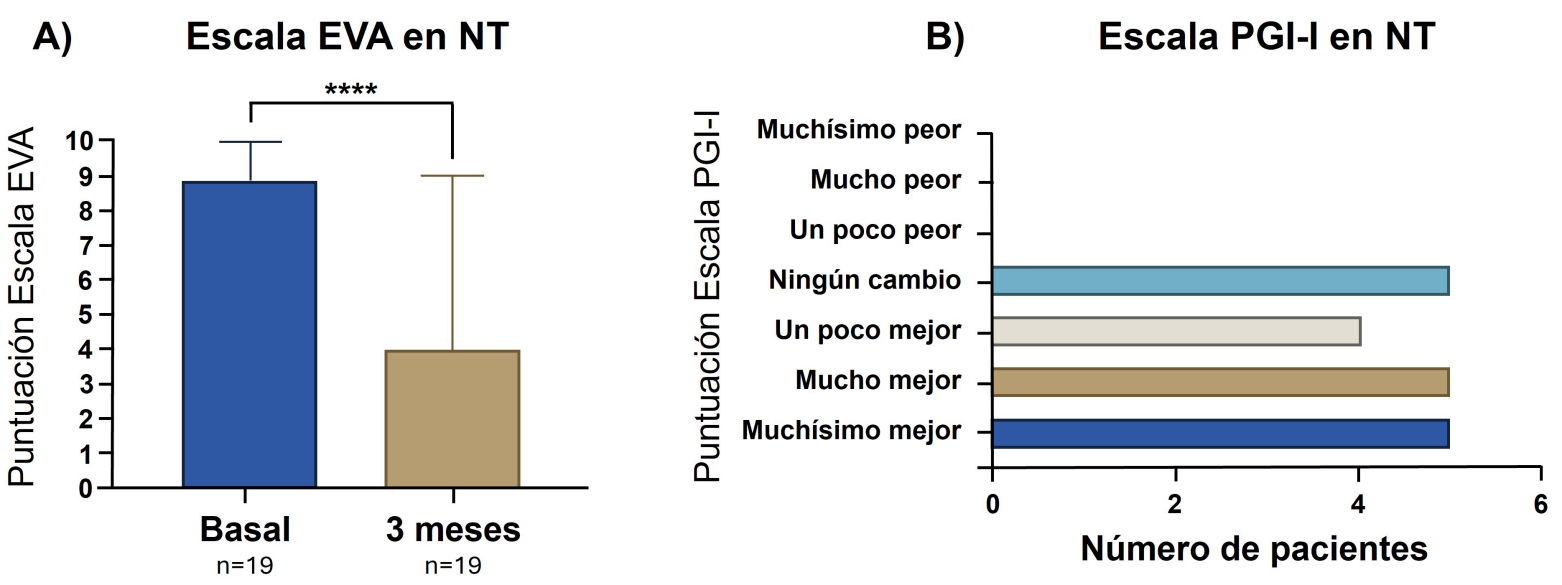
**Puntuación en la escala EVA antes y después de la RPP y puntuación en escala PGI-I a los 3 meses en pacientes con NT**. EVA, ecala vsual aalógica; PGI, *Patient Global Impression*. ****p < 0.0001. Test de estadística empleado: Wilcoxon test.

En relación con la escala EVA a los tres meses, se observó una mejoría en la puntuación en 16/19 (84,2%) pacientes. La puntuación en EVA previa a la radiofrecuencia fue ≥7 en todos los pacientes, incluso 9 pacientes (47,4%) presentaron un EVA de 10, y tras 3 meses del procedimiento solo era ≥7 en 6 pacientes (31,6%), e incluso 6 pacientes refirieron una puntuación EVA de 0. Las diferencias entre la puntuación EVA previa a la radiofrecuencia y tres meses tras la misma fueron estadísticamente significativas (*p* < 0,0001). En la Fig. 3A se representa la mediana de la puntuación EVA antes y después de la primera radiofrecuencia.

Respecto a la escala PGI-I a los tres meses, 5/19 pacientes (26,3%) mejoraron muchísimo, 5/19 (26,3%) mejoraron mucho, 4/19 (21,1%) mejoraron un poco y 5/19 (26,3%) no experimentaron ningún cambio respecto a su situación previa (Fig. 3B). Ningún paciente obtuvo una puntuación entre 5–7 tras la RPP.

De los pacientes que mejoraron, el dolor recurrió en 13/14 (92,8%). La recurrencia ocurrió en los primeros 3 meses tras la evaluación de efectividad (entre los 3 y 6 meses desde el procedimiento) en 2/13 pacientes (15,3%), entre 3 y 6 meses en 3/13 (23,1%), entre 6 meses y 1 año en 1/13 (7,7%) y pasado el año en 7/13 (53,8%). De los pacientes que recurrieron, 11/13 (84,6%) volvieron a la situación previa a la realización de la radiofrecuencia.

Finalmente, cabe mencionar que de los 7 pacientes que presentaban compresión vascular por resonancia magnética (RM) cerebral y que recurrieron tras la respuesta inicial a la radiofrecuencia, a 2/7 (28%) se les realizó una cirugía de microdescompresión vascular según la técnica de Janetta con mejoría posterior. De los restantes 5/7 (71,4%) con compresión vascular, 4/7 (57,1%) estaban bien controlados del dolor y 1/7 (14,2%) rechazó la cirugía.

#### 3.2.2 Cefalea en Racimos

**Fig. 4.**
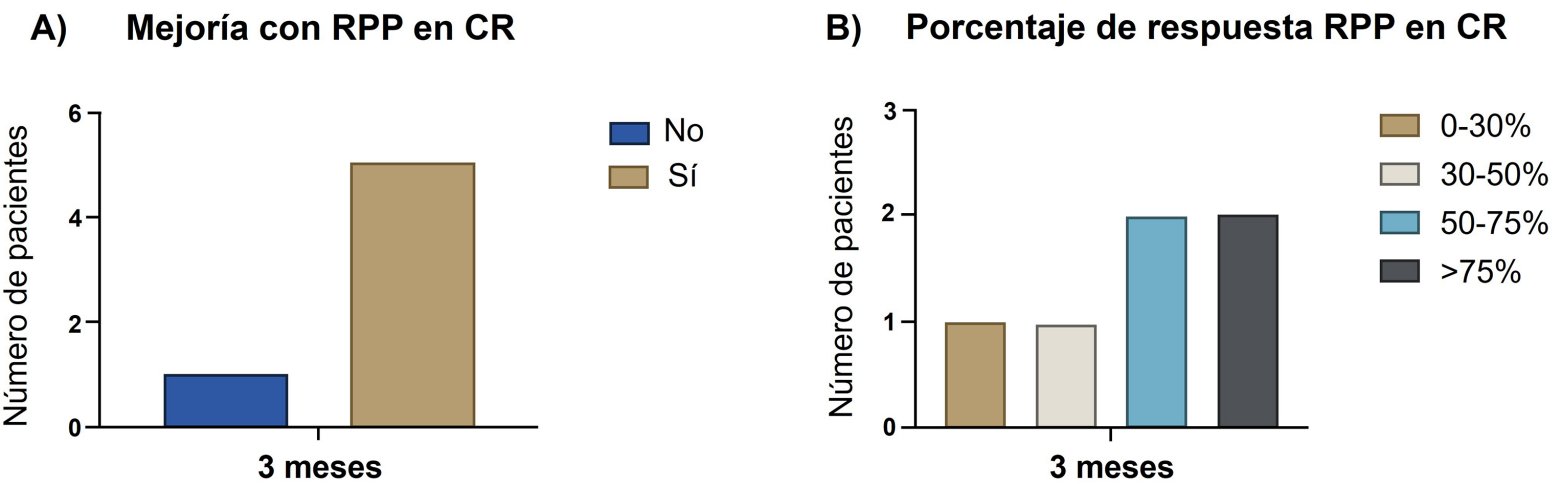
**Efectividad de la RPP en pacientes con CR**. (A) Pacientes que presentaron algún tipo de mejoría en el número de crisis. (B) Porcentaje de mejoría en el número de crisis mensuales. CR, cefalea en racimos.

Teniendo en cuenta el objetivo primario del estudio, mejoría en el número de ataques a los 3 meses, de los 6 pacientes, 5/6 (83,3%) presentaron algún grado de reducción tras la radiofrecuencia. El porcentaje de mejoría se incluye en la Fig. 4. Se alcanzó una tasa de respuesta de al menos 50% en 4/6 (66,7%) de los pacientes que mejoraron.

Además, hubo una reducción en el consumo de analgésicos en 5/6 pacientes (83,3%). El tiempo que tardó en evidenciarse la mejoría fue variable: 1/6 (16,6%) la presentó en menos de 72 horas, 4/6 (66,6%) tardaron entre 3 y 7 días y el paciente restante, 1/6 (16,6%), no mejoró.

**Fig. 5.**
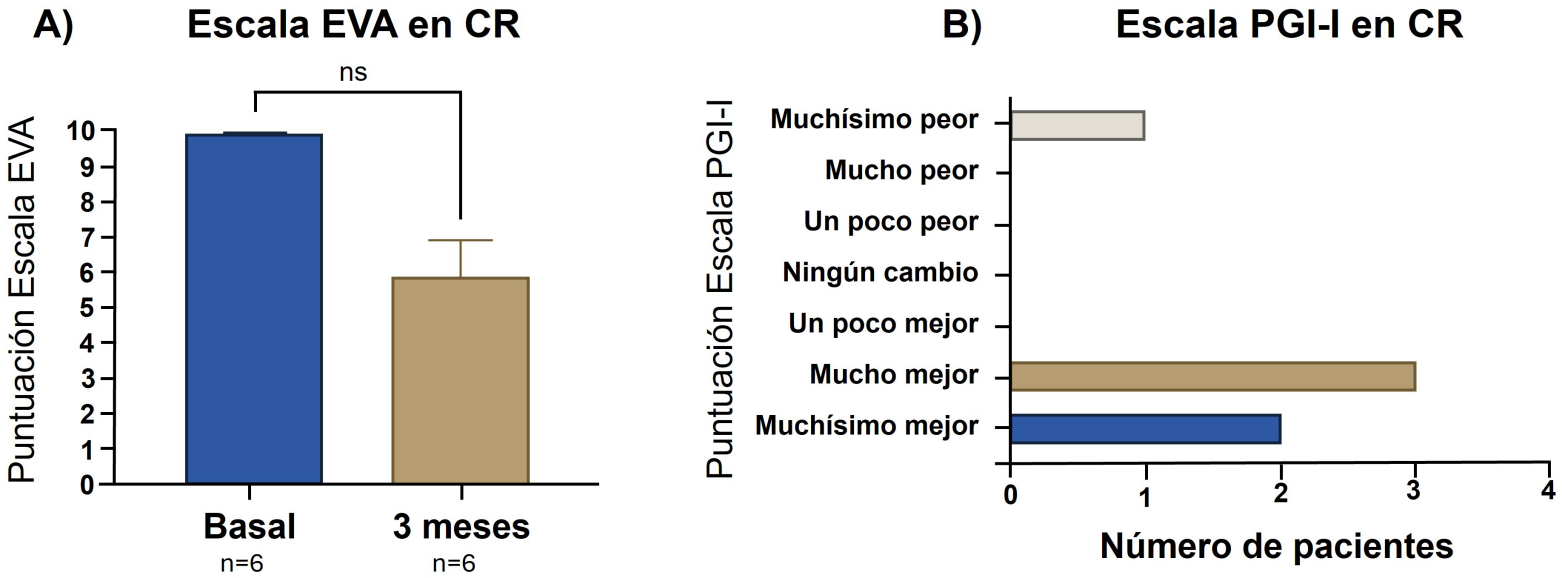
**Puntuación en la escala EVA antes y después de la RPP y puntuación en escala PGI a los 3 meses en pacientes con CR**. PGI, Patient Global Impression; Ns, no significancia estadística. Test de estadística empleado: Wilcoxon test.

En la valoración a los 3 meses la puntuación en la escala EVA disminuyó en 5/6 pacientes (83,3%). Antes de realizar el procedimiento, esta puntuación resultó ser ≥9 en todos los casos y a los 3 meses los pacientes otorgaron a su dolor las siguientes puntuaciones: 0 en 1/6 (16,6%), 2 en 1/6 (16,6%), 6 en 3/6 (50%) y 10 en 1/6 (16,6%). Esta reducción no resultó estadísticamente significativa (*p* = 0,06). En cuanto a la escala PGI-I a los 3 meses, 2/6 pacientes (33,3%) mejoraron muchísimo, 3/6 (50%) mejoraron mucho y 1/6 (16,6%) empeoró muchísimo. En la Fig. 5 se incluyen las puntuaciones de la escala EVA antes y después del tratamiento y las puntuaciones en la escala PGI-I a los 3 meses.

El dolor recurrió en los 5 pacientes (100%). La recurrencia tuvo lugar entre los 3 y 6 meses tras la radiofrecuencia en 2/5 pacientes (40%), entre 6 meses y 1 año en 1/5 pacientes (20%) y pasado el año en 2/5 pacientes (40%). De los pacientes que recurrieron, 4/5 (80%) volvieron a la situación previa a la radiofrecuencia. Finalmente, a 3/6 pacientes (50%) se les acabó implantando un neuroestimulador durante el seguimiento.

### 3.3 Seguridad del Procedimiento

No hubo ningún efecto adverso grave asociado a la técnica. Se reportaron dos efectos adversos leves en dos pacientes con NT: 1/25 (4%) presentó fotofobia y rinitis y 1/25 (4%) refirió parestesias que remitieron con el tiempo.

### 3.4 Asociación de Variables Clínicas y Respuesta a RPP

#### 3.4.1 Neuralgia del Trigémino

En el análisis exploratorio realizado entre los pacientes respondedores (mejoría >50%) y no respondedores (mejoría <50%) no encontramos diferencias estadísticamente significativas entre ambos grupos en relación con variables clínico-demográficas. La distribución de las variables en los dos grupos se incluye en la Tabla 2.

**Tabla 2.** **Variables clínicas y demográficas de los pacientes respondedores y no respondedores con neuralgia del trigémino tratados mediante RPP**.

Variables		No respondedor (N = 8)	Respondedor (N = 11)	*p*-valor
Mujeres, n (%)		5,0 (62,5)	8,0 (72,7)	0,645
Edad en años, media (DE)		76,4 (8,9)	76,7 (13,5)	0,950
HTA, n (%)		6,0 (75,0)	5,0 (45,5)	0,374
DL, n (%)		5,0 (62,5)	1,0 (9,1)	0,273
DM, n (%)		0 (0)	1,0 (9,1)	0,394
Hábito tabáquico, n (%)		1,0 (12,5)	1,0 (9,1)	0,816
Trastorno del ánimo, n (%)		1,0 (12,5%)	5,0 (45,5)	0,138
AP de otra patología que curse con dolor crónico, n (%)		0 (0%)	1,0 (9,1)	0,394
Edad de inicio de la enfermedad (años), media (DE)		67,1 (10,6)	63,6 (14,6)	0,574
Edad de diagnóstico de la enfermedad (años), media (DE)		68,0 (9,9)	64,7 (13,1)	0,280
Tiempo de evolución de la enfermedad en 1^a^ RF (años), media (DE)		4,9 (3,4)	9,3 (5,6)	0,065
Nº fármacos preventivos usados, media (DE)		5,0 (1,2)	4,2 (1,5)	0,214
Estudio de imagen realizado				
	TC craneal, n (%)	1,0 (12,5)	1,0 (9,1)	0,816
	RM craneal, n (%)	7,0 (87,5)	10,0 (90,9)	0,816
Presencia contacto vascular, n (%)		4 (57,1)	3,0 (30,0)	0,278
EVA basal, media (DE)		9,3 (1,2)	8,9 (1,0)	0,512
EVA 3 meses, mediana (RIQ)		9,0 (6,8–9,3)	0 (0–2,5)	<0,001**
PGI 3 meses, mediana (RIQ)		4,0 (3,0–4,0)	2,0 (1,0–2,0)	<0,001**
Reducción de analgesia, n (%)		3,0 (37,5)	9,0 (81,8)	0,074
Efectos adversos graves, n (%)		0 (0)	0 (0)	1

Respondedor = respuesta >50%; no respondedor: respuesta <50%. **p < 0,01. Estadística utilizada: prueba exacta de Fisher; prueba de chi-cuadrado de Pearson; U de Mann–Whitney, prueba *t* de Student; RIQ, rangos intercuartílicos. En “Presencia contacto vascular”, el valor de n es 7.

En relación con variables de respuesta y seguridad, no hubo diferencias en el EVA basal entre los pacientes respondedores y no respondedores. Se encontraron diferencias estadísticamente significativas en el EVA a los 3 meses entre los dos grupos (*p* < 0,001). Además, también se encontraron diferencias estadísticamente significativas en PGI-I (*p* < 0,001). No se observó una diferencia en la reducción del consumo de analgésicos. Tampoco hubo diferencias en la presencia de eventos adversos graves.

#### 3.4.2 Cefalea en Racimos

Tampoco encontramos diferencias estadísticamente significativas entre respondedores (mejoría >50%) y no respondedores en relación con las variables clínico-demográficas, o relación entre las variables de respuesta y las de seguridad. La distribución de las variables en los dos grupos se incluye en la Tabla 3.

**Tabla 3.** **Variables clínicas y demográficas de los pacientes respondedores y no respondedores con cefalea en racimos tratados mediante RPP**.

Variables		No respondedor (N = 2)	Respondedor (N = 4)	*p*-valor
Mujeres, n (%)		0 (0)	1 (25)	>0,9
Edad en años, media (DE)		35,5 (2,1)	60,5 (17,3)	0,7
HTA, n (%)		0 (0)	1 (25)	>0,9
DL, n (%)		0 (0)	1 (25)	>0,9
DM, n (%)		0 (0)	0 (0)	-
Hábito tabáquico, n (%)		1 (50)	2 (50)	>0,9
Trastorno del ánimo, n (%)		0 (0)	1 (25)	>0,9
AP de otra patología que curse con dolor crónico, n (%)		0 (0)	0 (0)	-
Edad de inicio de la cefalea (años), media (DE)		24 (8,5)	38 (16,9)	0,7
Edad de diagnóstico de la cefalea (años), media (DE)		29 (5,7)	42 (18,2)	>0,9
Tiempo de evolución de la cefalea en 1^a^ RF (años), media (DE)		7,5 (7,8)	16,75 (14,9)	0,7
Nº fármacos preventivos usados, media (DE)		7 (1,4)	4,75 (2,2)	>0,9
Estudio de imagen realizado				>0,9
	TC craneal, n (%)	0 (0)	2 (50)	
	RM craneal, n (%)	2 (100)	2 (50)	
EVA basal, mediana (RIQ)		9,5 (9,25–9,75)	10 (8,0–10,0)	>0,9
EVA 3 meses, media (DE)		8 (2,8)	3,5 (3)	0,5
PGI 3 meses, mediana (RIQ)		5 (4–6)	2 (1–3)	0,2
Reducción de analgesia, n (%)		1 (50)	4 (100)	0,14
Efectos adversos graves, n (%)		0 (0)	0 (0)	-

Respondedor = respuesta >50%; no respondedor: respuesta <50%. Estadística utilizada: prueba exacta de Fisher; U de Mann–Whitney; prueba *t* de Student.

## 4. Discusión

Nuestro estudio ha tenido como objetivo analizar de forma retrospectiva la efectividad y seguridad de la RPP en pacientes con NT y CR refractarias. De acuerdo con nuestros resultados, un alto porcentaje de pacientes presentó algún grado de mejoría tras el procedimiento, y una proporción relevante alcanzó una respuesta clínicamente significativa (≥50%), con mejoría en las escalas EVA y PGI-I, sin observarse efectos adversos de interés. No obstante, la mayoría de los pacientes presentó recurrencia del dolor durante el seguimiento, especialmente a partir del primer año tras el procedimiento, lo que pone de manifiesto la necesidad de considerar esta recurrencia al establecer expectativas realistas sobre la duración del efecto del tratamiento y al valorar la posibilidad de repetir el procedimiento en la práctica clínica habitual.

La evidencia disponible sobre la efectividad de la RPP es limitada, estando constituida principalmente por series de casos con un número reducido de pacientes, especialmente en el caso de la NT. Además, los datos sobre recurrencia tras el procedimiento son escasos [2, 17]. En nuestro estudio se presenta una serie de 19 pacientes con NT, que, aunque de tamaño limitado, se encuentra en línea con las series previamente publicadas y aporta información adicional sobre la evolución clínica y el riesgo de recurrencia tras la RPP en la práctica clínica real.

### 4.1 Neuralgia del Trigémino

Nuestro estudio encontró que la RPP logró una mejoría en el número de días con dolor en 73,6% pacientes, siendo >50% en 57,9%, y una reducción del consumo de analgésicos en 63,1% pacientes. En línea con lo descrito en la bibliografía previa [18, 19, 20], siendo una de las series con mayor tamaño muestral, confirmamos la efectividad de la RPP en el tratamiento de esta patología. Además, encontramos diferencias estadísticamente significativas (*p* < 0,001) entre las puntuaciones que los pacientes otorgaron a su dolor en la escala de intensidad del dolor EVA pre y post-radiofrecuencia.

De los 14 pacientes que mejoraron, el dolor recurrió en 92,8%, lo que sugiere una alta tasa de recurrencia a pesar de la buena respuesta inicial, similar a lo descrito hasta el momento (80%) [2].

A pesar de la alta tasa de recurrencia, la mayoría de los pacientes obtuvo un beneficio significativo tras el procedimiento. Se ha descrito una resistencia al tratamiento en un 25–30% de los pacientes con NT [13]. Teniendo en cuenta la importante discapacidad que el dolor ocasiona en estos casos, especialmente en pacientes refractarios a los tratamientos disponibles, el impacto de la RPP en la calidad de vida de los pacientes es muy destacable, sobre todo considerando que la mejoría, a pesar de ser transitoria, persistió en un 54% durante más de un año. Por otro lado, dado que la técnica es segura, tras la recurrencia de dolor se pueden realizar sucesivos procedimientos.

Siendo una técnica plausible, mínimamente invasiva, segura y efectiva, y no habiendo identificado en nuestro estudio factores predictores de respuesta, consideramos que podría plantearse en pacientes con NT clásica refractaria tras valorar el balance riesgo-beneficio de forma individualizada. Sería una estrategia a considerar en pacientes resistentes a los tratamientos farmacológicos habituales para reducir la inercia terapéutica y mejorar la calidad de vida [21], en aquellos con alto riesgo quirúrgico, edad avanzada o que rechazan la intervención quirúrgica, en casos sin contacto vascular claro en la neuroimagen (teniendo en cuenta las limitaciones inherentes a estas técnicas), o en formas con dolor facial persistente concomitante.

### 4.2 Cefalea en Racimos

Nuestro estudio ha mostrado una disminución en el número de ataques y en el consumo de analgésicos en más del 80% de los pacientes incluidos con CRCR. La mejoría fue superior al 50% en 66,7%. Estos resultados apoyan la efectividad de esta técnica en este tipo de dolor craneofacial [6, 17, 22], si bien, y a pesar de lograr una reducción en las crisis de dolor, no se encontraron diferencias estadísticamente significativas en la escala EVA pre y post procedimiento, probablemente por el pequeño tamaño muestral.

El dolor recurrió en todos los pacientes, teniendo lugar esta recurrencia, transcurrido al menos un año del procedimiento en 2 de los 5 pacientes (40%).

En pacientes con CRCR, al igual que en NT, tampoco encontramos factores predictores de respuesta a RPP.

### 4.3 Seguridad

Respecto a la seguridad, no se reportaron efectos adversos graves asociados a la técnica en ninguno de los casos. Nuestros resultados coinciden con los de estudios previos, en los que los pacientes no mostraron complicaciones o estas fueron leves, a diferencia de la RFC en la que se han descrito mayores complicaciones, entre ellas, hipoestesia, disestesia y pérdida de fuerza del masetero [2, 6, 23, 24].

Entre las limitaciones del presente estudio se encuentran su carácter retrospectivo y el reducido tamaño muestral, especialmente en CR, requiriendo confirmación en estudios prospectivos con mayor tamaño muestral. La naturaleza retrospectiva del diseño dificulta garantizar una cuantificación precisa y homogénea de la reducción del dolor, especialmente en lo relativo al número de días con dolor o a la frecuencia de ataques, al basarse en registros clínicos y en la información referida por los pacientes. Cabe señalar, además, que la neuroimagen puede no detectar contactos neurovasculares sutiles incluso cuando se realiza con protocolo adecuado. Por otro lado, la información disponible en los registros clínicos no permitió diferenciar de forma sistemática a los pacientes con dolor exclusivamente paroxístico de aquellos que podrían presentar un componente de dolor facial persistente concomitante, distinción que podría tener implicaciones en la respuesta al tratamiento y en la tasa de recurrencia, y que debería recogerse de forma prospectiva en futuros estudios. Por ello, son necesarios estudios prospectivos, con muestras más amplias, seguimiento a largo plazo y herramientas estandarizadas de registro del dolor, que permitan evaluar de forma más precisa la efectividad de la técnica e identificar posibles factores predictores de respuesta.

No obstante, estos resultados deben interpretarse con cautela debido al carácter retrospectivo del estudio y al tamaño muestral limitado, especialmente en el subgrupo de CR, Si bien no se ha logrado determinar factores clínicos y/o sociodemográficos de respuesta y un porcentaje elevado de pacientes mostró recurrencia del dolor, la mayoría experimentó un beneficio inicial significativo. Esta información es relevante para gestionar las expectativas sobre la durabilidad del efecto de la RPP en la práctica clínica y para planificar posibles procedimientos repetidos. Dado el adecuado balance riesgo-beneficio, además de su bajo coste, consideramos que la RPP es una técnica que podría considerarse de forma individualizada en casos refractarios de NT o CRCR.

## 5. Conclusiones

La RPP es una técnica terapéutica alternativa efectiva y segura en pacientes con neuralgia del trigémino y cefalea en racimos crónicas refractarias, asociada a un beneficio clínico inicial relevante pese a la frecuente recurrencia del dolor. Su bajo perfil de complicaciones y su carácter mínimamente invasivo respaldan su posible papel dentro del abordaje de pacientes con opciones limitadas o no candidatos a tratamiento quirúrgico. Estos resultados deben interpretarse con cautela por el diseño retrospectivo y el tamaño muestral limitado, siendo necesarios estudios prospectivos que confirmen estos hallazgos.

## Data Availability

Los datos presentados en este estudio están disponibles previa solicitud al autor de corresponden cia, debido a restricciones éticas y legales relacionadas con la protección de información clínica sensible. Los datos fueron anonimizados y almacenados en una base de datos para su posterior análisis estadístico. Estos datos solo po drán compartirse con profesionales sanitarios cualificados e investigadores especializados en el ámbito de la cefalea que acepten cumplir las condiciones institucionales de uso de datos y justifiquen adecuadamente su necesidad.

## Abreviaturas

RPP, radiofrecuencia pulsada percutánea; RFC, radiofrecuencia convencional; NT, neuralgia del trigéminol; CR, cefalea en racimos; CRCR, cefalea en racimos crónica y refractaria; GSP, ganglio esfenopalatino; ICHD-3, tercera edición clasificación internacional cefaleas.

## Contribuciones de los Autores

MD y ABGV diseñaron el estudio. MD y CM redactaron el manuscrito. MD, AGM y IFL realizaron el análisis de los datos. MD, AGM, SQ, CM, ER, MM, DO, CP, JV y ABGV llevaron a cabo la recogida de datos. ABGV y JV revisaron el manuscrito. Todos los autores contribuyeron a los cambios editoriales en el manuscrito. Todos los autores leyeron y aprobaron la versión final del manuscrito. Todos los autores han participado suficientemente en el trabajo y han aceptado ser responsables de todos los aspectos del mismo.

## Aprobación Ética y Consentimiento Informado

El estudio fue aprobado por el comité de ética del Hospital Universitario de la Princesa (número de registro 4690). Todos los pacientes firmaron el consentimiento informado por escrito. El estudio se realizó conforme a los principios de la Declaración de Helsinki.

## Financiación

Esta investigación no recibió financiación externa.
